# *Bacillus subtilis* and *Enterococcus faecium* co‐fermented feed regulates lactating sow's performance, immune status and gut microbiota

**DOI:** 10.1111/1751-7915.13672

**Published:** 2020-10-07

**Authors:** Cheng Wang, Siyu Wei, Bocheng Xu, Lihong Hao, Weifa Su, Mingliang Jin, Yizhen Wang

**Affiliations:** ^1^ Key Laboratory of Molecular Animal Nutrition Ministry of Education Zhejiang University 866 Yuhang Tang Road Hangzhou Zhejiang 310058 China; ^2^ Key Laboratory of Animal Nutrition and Feed Ministry of Agriculture Zhejiang University 866 Yuhang Tang Road Hangzhou Zhejiang 310058 China; ^3^ National Engineering Laboratory of Biological Feed Safety and Pollution Prevention and Control Zhejiang University 866 Yuhang Tang Road Hangzhou Zhejiang 310058 China; ^4^ Key Laboratory of Animal Nutrition and Feed Science of Zhejiang Province Zhejiang University 866 Yuhang Tang Road Hangzhou Zhejiang 310058 China; ^5^ College of Animal Science Institute of Feed Science Zhejiang University 866 Yuhang Tang Road Hangzhou Zhejiang 310058 China

## Abstract

Fermented feed (FF) is widely applied to improve swine performance. However, the understandings of the effects of FF on the immune status and gut microbiota of lactating sows and whether probiotics are the effective composition of FF are still limited. The present study aimed to investigate the performance, immune status and gut microbiota of lactating sows fed with a basal diet supplemented with *Bacillus subtilis* and *Enterococcus faecium* co‐fermented feed (FF), with the probiotic combination (PRO) of *B. subtilis* and *E. faecium* and control diet (CON) as controls. Compared with the CON group, FF group remarkably improved the average daily feed intake of sows and the weight gain of piglets, while significantly decreased the backfat loss, constipation rate of sows and diarrhoea incidence of piglets. The yield and quality of milk of sows in FF group were improved. Besides, faecal acetate and butyrate were promoted in FF group. Additionally, FF increased the level of IgG, IgM and IL‐10 and decreased the concentration of TNF‐α in serum. Furthermore, FF reduced the abundance of *Enterobacteriaceae* and increased the level of *Lactobacillus* and *Succiniclasticum*, which were remarkably associated with growth performance and serum immune parameters. Accordingly, microbial metabolic functions including DNA repair and recombination proteins, glycolysis and gluconeogenesis, mismatch repair and d‐alanine metabolism were significantly upregulated, while amino acid metabolism was downregulated in FF group. Overall, the beneficial effects of FF were superior to PRO treatment. Altogether, administration of FF during lactation improved the performance and immune status, and modulated gut microbiota of sows. Probiotics are not the only one effective compound of FF.

## Graphical abstract

This study demonstrates the beneficial effects of *Bacillus subtilis* and *Enterococcus faecium* co‐fermented feed on the performance, immune status and gut microbiota of lactating sows and firstly clarifies probiotics are not the only one effective factors in FF, which expanding the knowledge of underlying mechanism of FF’s beneficial effects.

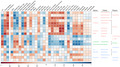

## Introduction

Fermentation plays important roles not only in human food but also in animal husbandry feed. Overuse of antimicrobial growth promoters in animal husbandry can lead to serious problems such as superbugs generation, residues in animal products and environmental pollution (Butaye *et al*., 2003). Solid‐state fermented feed (FF) contains superior nutritional quality and low antinutritional factor (ANF) content and is considered as one of the promising alternatives to the antimicrobial growth promoters (Wang *et al*., 2018b). Inoculated microbes determine the quality of FF. Aerobic microbes like *Bacillu*s spp. can secrete abundant extracellular enzymes to degrade ANFs and greatly change the physicochemical characteristics of substrates (Plumed‐Ferrer and Wright, 2009; Kiarie *et al*., 2011; Shi *et al*., 2017). Lactic acid bacteria like *Lactobacillus* spp. are good at organic acid production under anaerobic environment (Missotten *et al*., 2015). Additionally, the inoculated probiotics can improve the micro‐ecological environment of feed (Wang *et al*., 2020).

Supplementing with FF has been dramatically investigated to improve the feed digestibility, balance intestinal microbiota, stay gut health and modulate immunity, thereby benefiting swine health and performance (Canibe and Jensen, 2003; Missotten *et al*., 2015; Rahman *et al*., 2015). Lactation period of sows is an important stage of swine breeding (Tokach *et al*., 2019). Several studies reported the beneficial effects of FF on the performance of lactating sows and their offspring (Demeckova *et al*., 2002; Shen *et al*., 2011; Wang *et al*., 2018a). However, whether FF ameliorates the immune status and gut microbiota of lactating sows and the effective composition of FF is probiotics remains unclear.

Therefore, the present study was conducted to investigate the effects of *Bacillus subtilis* and *Enterococcus faecium* co‐fermented feed on the performance, immune status and gut microbiota of lactating sows and determine whether the inoculated probiotics were effective factors of FF.

## Results

### Nutritional composition

Table 1 shows the nutrition composition of the unfermented feed (UF) and FF. The UF contained lower content of crude protein (CP), ash and total phosphorus (P) in contrast to the FF. However, ethanol extract (EE) was at a lower level in the FF than that in the UF. The concentration of trichloroacetic acid–soluble protein (TCA‐SP) in the UF was 3.57%, while the content increased to 14.61% in FF. The fermentation process resulted in the degradation of 36.93% of neutral detergent fibre (NDF), 23.32% of acid detergent fibre (ADF), 57.10% of amylose and the majority of antigenic proteins in the UF. Also, the number of *B. subtilis* and *E. faecium* increased to 5.3 × 10^8^ and 4.7 × 10^8^ respectively. 164.38 mmol kg^−1^ of higher lactic acid level and 4.62 of lower pH in the FF were observed.

**Table 1:** Nutrient composition of fermented mixed feed (as‐fed basis).^a^

Item	UF^b^	FF^c^
DM, %	91.34	90.26
CP, %	26.56	29.19
TCA‐SP, %	3.57	14.61
NDF, %	14.73	9.29
ADF, %	7.46	5.72
Amylose, %	5.10	2.19
EE, %	3.67	3.37
Ash, %	3.89	4.17
Ca, %	0.19	0.18
Total P, %	0.46	0.53
β‐conglycinin, mg g^−1^	26.54	4.66
Glycinin, mg g^−1^	57.47	8.46
pH	6.67	4.62
Lactic acid, mmol kg^−1^	–	164.38
Live BS cells, cfu g^−1^	–	5.3 × 10^8^
Live EF cells, cfu g^−1^	–	4.7 × 10^8^

BS, *Bacillus subtilis*; EE, ether extract; EF, *Enterococcus faecium*; TCA‐SP, trichloroacetic acid‐soluble protein (small peptides).

^a^ Analysed values determined in duplicate.

^b^ UF = unfermented mixed feed (40% corn, 40% soybean meal and 20% yellow wine lees).

^c^ FF = fermented mixed feed (40% corn, 40% soybean meal and 20% rice distiller’s dried grains with soluble.)

### Sows’ lactating performance and faecal short‐chain fatty acids (SCFAs)

As shown in Table 2, the numbers of piglet total born, live born and at weaning had no differences in the groups. A significant upward trend in ADFI (*P* < 0.05) and a decreased in the backfat loss (*P* < 0.05) and constipation rate were observed in the FF group (*P* < 0.05) in contrast with the CON. Fermented feed significantly increased the piglet weaning weight and bodyweight gain (*P* < 0.05). Moreover, dietary FF treatment remarkably decreased the piglet diarrhoea incidence (*P* < 0.05) in contrast with that in the CON. Compared with the CON, adding 10% of FF showed significant improvement in milk yield, milk fat and milk protein and decreased the amount of sows’ urea nitrogen (N) and somatic cell count (SCC) (*P *< 0.05).

**Table 2:** Effects of inclusion of 10% fermented mixed feed and probiotics on the performance of the lactating sows and piglets.

Item	Diet			SEM	*P*‐value
	Control	10% FF	Probiotic		
Sow					
ADFI,^1^ kg d^−1^	5.64^b^	6.08^a^	5.73^b^	0.07	0.01
Backfat loss,^2^ mm	2.01^a^	1.39^b^	1.58^b^	0.08	0.00
Constipation rate, %	5.90^a^	5.45^b^	5.47^b^	0.08	0.07
Piglets					
Number at birth, total	12.00	12.33	11.67	0.31	0.71
Number at birth, live	11.67	12.00	11.50	0.25	0.73
Number at weaning	11.33	11.67	11.00	0.24	0.56
Weaning alive rate,^3^ %	97.22	97.33	95.77	0.99	0.79
Wt at birth,^4^ kg	1.23	1.21	1.22	0.01	0.62
Wt at weaning,^5^ kg	5.36^b^	5.69^a^	5.40^b^	0.06	0.05
Wt gain,^6^ kg	4.14^b^	4.48^a^	4.27^b^	0.06	0.04
Diarrhoea incidence,^7^ %	1.48^a^	1.12^c^	1.24^b^	0.04	0.00
Milk					
Yield, kg	8.60^b^	9.02^a^	8.80^b^	0.07	0.03
Fat, %	6.69^b^	7.22^a^	6.83^ab^	0.10	0.06
Lactose, %	6.05	6.04	5.97	0.04	0.70
Protein, %	4.44^b^	4.94^a^	4.89^a^	0.10	0.08
Solids, %	17.68	17.73	17.67	0.22	0.99
Urea N, mmol l^−1^	53.60^a^	50.38^b^	49.27^b^	0.84	0.09
SCC, 10^3^ per l	1099.67^a^	746.83^b^	899.83^b^	59.64	0.04

^a,b^Means within a row with different superscripts significantly differ (*P* < 0.05).

SCC, somatic cell counts. All the values contained six repetitions.

^1^ ADFI of the sows was recorded from parturition until weaning (21 days).

^2^ Backfat loss = parturition backfat − weaning backfat.

^3^ Weaning alive rate = [litter size at weaning (live) − litter size at birth (live)]/litter size at birth (live).

^4^ Piglet weight at birth = litter weight at birth/litter size at birth (live).

^5^ Piglet weight at weaning = litter weight at weaning/litter size at weaning (live).

^6^ Piglet weight gain = piglet weight at weaning − piglet weight at birth.

^7^ Diarrhoea incidence = total diarrhoea piglets/[litter size at birth (live) × trial days].

**Fig. 1:**
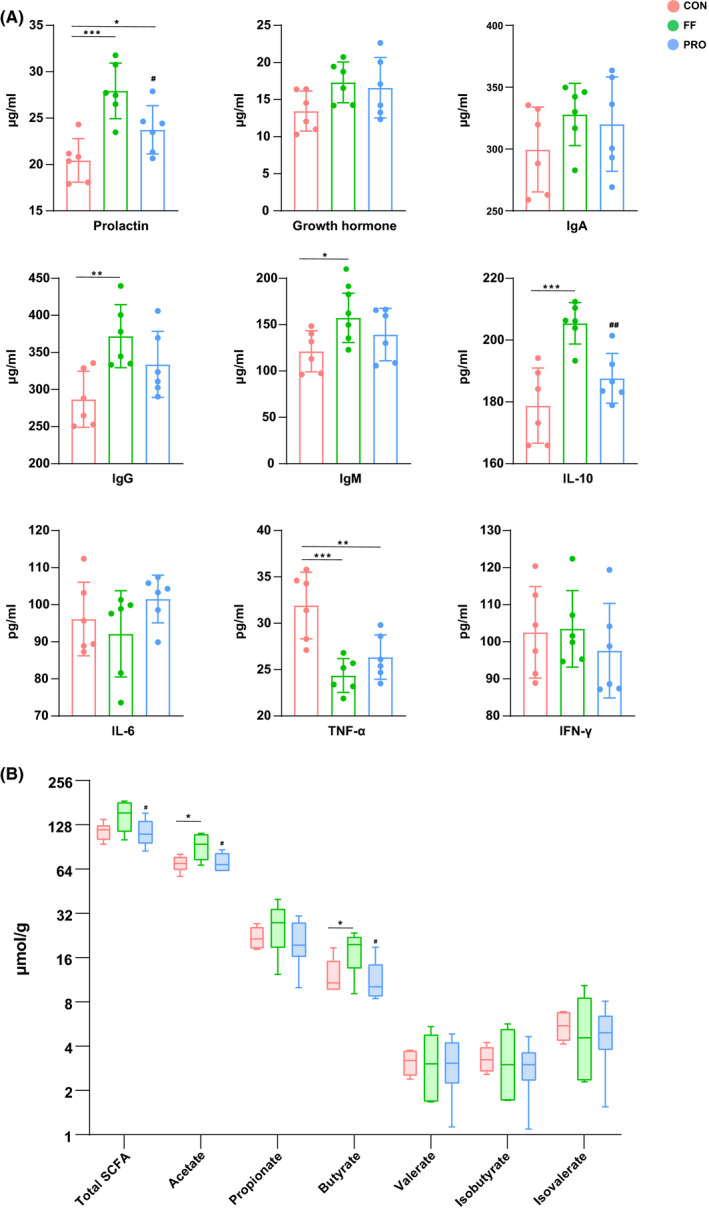
Serum indexes and faecal SCFA content of lactating sows. A. Serum prolactin, growth hormone, IgA, IgG, IgM, IL‐10, IL‐6, TNF‐α and IFN‐γ. B. Faecal total SCFA, acetate, propionate, butyrate, valerate, isobutyrate and isovalerate. *P < 0.05, **P < 0.01 and ***P < 0.001 compared with the CON. #*P* < 0.05 and ##*P* < 0.01 for PRO vs. FF. All the values contained six repetitions.

Short‐chain fatty acid content in the faeces of lactating sows fed FF or PRO is shown in Figure 1B. The PRO had no noteworthy effects on concentrations of SCFAs, comparing with the CON. The content of faecal acetate and butyrate was greatly improved in the FF group than those in CON and PRO (*P* < 0.05). Additionally, total SCFAs tended to increase after feeding FF (*P* = 0.053).

### Serum parameter

Serum indexes were determined to assess the impact of FF and probiotics on sow’s physiological status and immunity. Results indicated that the level of serum prolactin was dramatically upregulated by both FF and PRO (Fig. 1A). Additionally, adding FF significantly promoted sows’ serum growth hormone. No differences were found at IgA content among the groups. Probiotics treatment promoted IgG content, while FF remarkably increased the level of both serum IgG and IgM (*P *< 0.05). For serum cytokines, dietary FF or PRO supplementation significantly improves the level of IL‐10 and reduces the content of TNF‐α (*P *< 0.05).

### Gut bacteria diversity and network

**Fig. 2:**
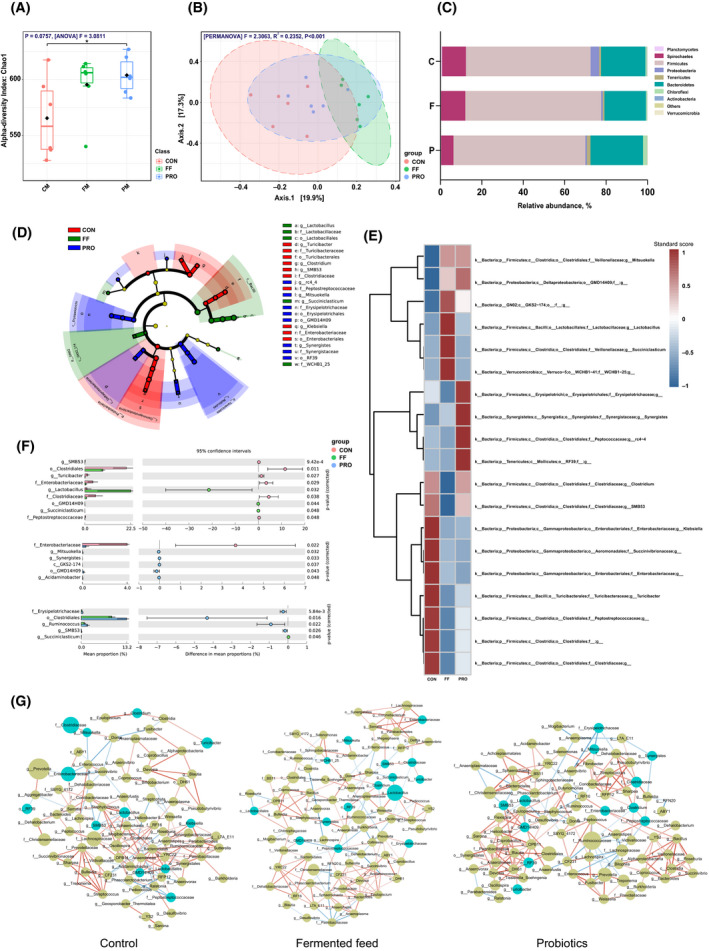
Description of gut microbial diversity. A. α diversity of gut bacteria based on Chao1 index. B. PCA plot of β‐diversity based on Bray–Curtis index. C. Bar plots at phylum level. D. Heatmap of distinguished genera among the groups. E. Cladogram of distinguished genera. F. FDR correction of significantly different genera. G. Network analysis of the 3 group by Pearson correlation. The correlation score > 0.8 or < −0.8 was selected. Red line represents positive relationships. Blue line represents negative relationships. The circle size indicates the relative abundance of genera. Circles in bottle green: distinguished genera. Circles in reseda: undistinguished genera. All the values contained six repetitions.

The diversity of gut bacteria tended to improve in the FF group (*P* = 0.084) and significantly increased in PRO group (*P* = 0.032, Fig. 2A) compared to that of the CON, indicating by the Chao1 index. No notable differences were found between the FF group and PRO group. Figure 2B shows the principal coordinate analysis (PCoA) and demonstrated that gut bacteria showed obvious shifts (*P* < 0.001) from the CON to FF and PRO [*P* < 0.001 by Bray–Curtis index and permutation MANOVA (PERMANOVA)]. Furthermore, the taxonomy bar plot revealed that *Firmicutes*, *Bacteroidetes*, *Spirochaetes* and *Proteobacteria* are the prevailing bacteria in sow’s intestinal microbiota (Fig. 2C). *Proteobacteria* significantly decreased in the FF‐treated group in contrast to CON (*P *＜ 0.05). *Lentisphaerae* and *Proteobacteria* significantly decreased in PRO group, while *Tenericutes* remarkably increased. Furthermore, the cladogram plot of LEfSe analysis (LDA > 2) was applied to find the distinct bacteria and the results showed obvious differences in microbial composition in three groups (Fig. 2D). Specifically, *Turicibacter*, *Klebsiella* and *Clostridium* showed a dramatic reduction in both the FF and PRO groups. The heatmap at the genera level shows the distinguishing bacteria abundance in each group (Fig. 2E). *Lactobacillus* and *Succiniclasticum* showed a remarkable increase in the FF group. Probiotics treatment improved the abundance of *Mitsuokella*, *Synergistes*. The results of distinguishing genera were further verified by statistical analysis of metagenomic profiles (STAMP) multiple‐test correction (Fig. 2F).

To further study the correlation of microbiota in groups, network analysis was performed using Pearson’s correlation coefficients. The results demonstrated that FF inclusion regulated interactions (Fig. 2G). The gut microbiota influenced by FF treatments had less cross‐linking and shorter interactions indicated by lower plot densities (0.0050 and 0. 0047 in the CON and FF groups, respectively), lower network diameters (11 and 10), lower network heterogeneity (0.60 and 0.48) and higher cluster coefficients (0.29 and 0.32) in contrast with CON. Similarly, PRO had lower network heterogeneity (0.48) and higher cluster coefficients (0.32) compared with the CON. However, the plot densities were increased in PRO (0.051).

### Metagenomic functional predications

**Fig. 3:**
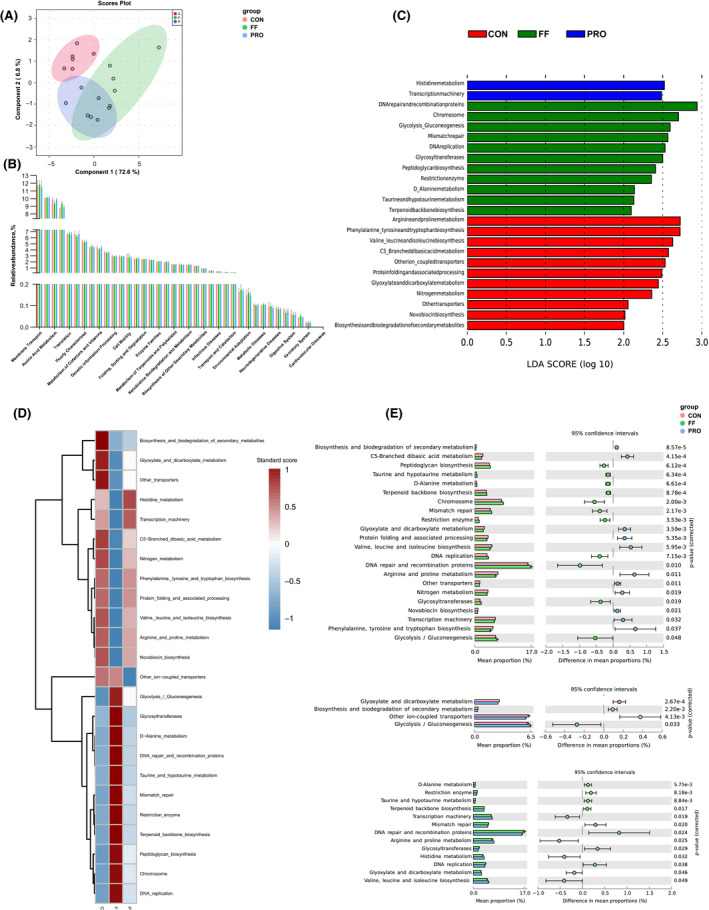
Metagenomic functional predications. A. The sparse PLS‐DA algorithm of predicated metabolic functions. B. Bar plots of bacterial functions at level 2. C. LEfSe result of distinguished microbial functions at level 3. D. Heatmap of significantly different microbial functions. E. Statistic corrections of distinguished functions by STAMP. All the values contained six repetitions.

Phylogenetic investigation of communities by reconstruction of unobserved states (PICRUSt) was further used to obtain the predicted metabolic functions of gut microbiota. The sPLSDA plot showed that the bacterial functions indicated distinctive differences among the groups (Fig. 3A). Additionally, the findings suggested that 23 pathways at KEGG level 3 notably differed among groups including metabolism and genetic information processing. The significantly differed pathways were obtained based on LEfSe analysis (Fig. 3B). Also, DNA repair and recombination proteins, glycolysis and gluconeogenesis, mismatch repair and d‐alanine metabolism that were associated with replication and repair were significantly upregulated in the FF‐treated group (Fig. 3C). The heatmap showed the distribution of distinguishing functional pathways among each group (Fig. 3D). The STAMP analysis further verified the differences in metabolic functions (Fig. 3E).

### Correlation of feed characteristics, sow performance, serum parameters and gut microbiota

**Fig. 4:**
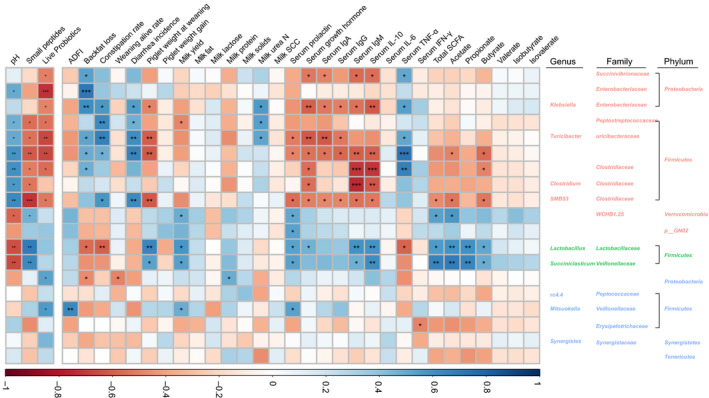
Correlation of gut bacteriome and feed characteristics, sow’s performance, serum indexes and SCFAs. The colour of genera is represented by red: CON group, green: FF group, blue: PRO group. Significant correlation is represented by ***P < 0.001, **0.001 < *P* < 0.01, *0.01 < *P* < 0.05 respectively. All the values contained six repetitions.

Pearson’s correlation was further analysed to investigate the relationship among gut microbiota, apparent indicators of FF and sows’ performance (Fig. 4). As to feed characteristics, *Lactobacillus* and *Succiniclasticum* enriched in FF were positively correlated with small peptides and negatively with pH (*P* < 0.01). While the genera most found in the CON, like *Turicibacter, Clostridium* and *SMB53,* showed opposite results (*P* < 0.05). *Mitsuokella* and GMD14H09, which were abundant in the PRO group, were positively related to live probiotic amount (*P* < 0.05).

As to sow performance, the gut bacteria in sows of CON were positively corrected with backfat loss, constipation rate, milk urea N and serum content of TNF‐α and were negatively correlated with litter weight gain, milk yield, serum hormone, serum immunoglobulin, serum inflammatory cytokines and some faecal SCFAs (*P* < 0.05). Fermented feed enriched *Lactobacillus* and *Succiniclasticum* had a positive relationship with litter weight gain, milk yield, serum prolactin, serum IgM, serum IL‐10, total SCFAs, acetate, propionate and butyrate (*P* < 0.05). Interestingly, *Lactobacillus* also had a significantly negative correlation with backfat loss, constipation rate, serum TNF‐α (*P* < 0.05). In PRO group, *Mitsuokella* in faeces of sows was positively corrected with ADFI, milk yield and serum prolactin (*P* < 0.05). *Erysipelotrichaceae* were negatively related to backfat loss and IFN‐γ (*P* < 0.05).

## Discussion

In the present study, a novel FF, *Bacillus subtilis* and *Enterococcus faecium* co‐fermented feed improved the performance of lactating sows and their offspring. Additionally, compared with the CON, FF modulated sows’ immune status and gut microbiota, which positively related with sows’ performance. Interestingly, the beneficial effects of FF are better than PRO.

Many studies have investigated the beneficial effects of FF on swine production (Feng *et al*., 2007; Canibe *et al*., 2008; Mukherjee *et al*., 2016; Wang *et al*., 2018c). However, due to the various fermenting substrates, inoculations and product characteristics among studies, the positive effects of FF on the performance of lactating sows are sometimes inconclusive. Here, we chosen the most commonly used feed ingredients corn, soybean meal (SBM) and one of the Chinese local feed ingredients yellow wine lees as the fermented substrates. To effectively degrading antinutritional factors (ANFs) and macromolecular nutrients and efficiently reduced the pH in the substrates, the two most common and effective fermenting probiotics, *B. subtilis* and *E. faecium,* were used (Chi and Cho, 2016). Therefore, these substrates and probiotics were combined and then a novel type of FF was obtained. Additionally, lactating sows were fed FF or the same amount of fermenting probiotics to further explore whether probiotic is the main effective factor of FF as FF is a complex mixture that is hard to distinguish the principal effective component.

Our results indicated that the FF contained more content of CP than that of the UF. Also, FF showed an improvement in TCA‐SP in contrast with that of UF. Crude protein, TCA‐SP, ANF, pH and probiotic count are important to assessing evaluation indicators for final fermented products (Missotten *et al*., 2010). Trichloroacetic acid‐soluble protein contains small molecular peptides that can be easily utilized in the intestine and plays antioxidant and immune regulation functions (Gilbert *et al*., 2008). 78.0% and 86.7% of β‐conglycinin and glycinin contents in UF were reduced after fermentation respectively. *B. subtilis* fermentation can degrade antigenic protein and trypsin inhibitors to increase the bioavailability of SBM (Seo and Cho, 2016). Thus, the hydrolysis of macromolecular proteins (including antigenic proteins) may contribute to the increase of TCA‐SP. Additionally, FF contained a large content of organic acids and abundant probiotics. Hence, not only a lower ANF content, greater CP and small peptides concentration existed in the FF‐included pellet feed in comparison to the UF, but also abundant live probiotic cells and their beneficial metabolites were supplied to sows.

The increased ADFI in sows, weaning weight and bodyweight gain in piglets was observed with 10% FF supplementation. Meanwhile, FF notably reduced the backfat loss, constipation rate of lactating sows and piglet diarrhoea incidence. Demeckova *et al*. (2002) reported that fermented liquid feed by *Lactobacillus* spp. is capable of improving lactating sow ADFI. Yun *et al*. (2019) demonstrated that dietary inclusion 0.1–0.2% fermented garlic improved the sow and litter performance. Xue *et al*. (2011) reported that adding 5% of fermented potato pulp positively affects sow and litter performance. These evidences indicate that FF has consistent beneficial effects on performance of sow and piglets. In comparison with the CON, backfat loss of sows and diarrhoea incidence of piglets were remarkably decreased in the PRO. Numerical improvements of ADFI and piglet weight gain were also found in the PRO group in comparison with the CON. Menegat *et al*. (2019) reported supplementing a probiotic *B. subtilis* C‐3102 to lactating sows did not show a notable increase in performance. Another study showed that providing probiotic *Enterococcus faecium* to lactating sow had no significant effect on litter growth performance (Taras *et al*., 2006). With the inclusion of *Bacillus mesentericus*, *Enterococcus faecalis* and *Clostridium butyricum,* the performance of sows and offspring was improved (Hayakawa *et al*., 2016). Probiotic treatment only significantly reduced the backfat loss of sows and diarrhoea incidence of piglets (*P* < 0.05). These evidences showed that the performance of sows could be influenced by the combinations of probiotics, which may not be affected by supplementing probiotics alone. Interestingly, the multi‐effects of FF on the performance of lactating sows generally are greater than that of PRO. It can be speculated that dietary inclusion of FF not only exerts probiotic roles of the inoculates, but also provides their metabolites such as organic acids, functional oligosaccharides, antimicrobial peptides and digestive enzymes which may exert the benefits observed here (Majumdar and Bose, 1958; Kim *et al*., 2007; Gao *et al*., 2012; Sriphannam *et al*., 2012). Besides, the composition of the novel substrate and proper supplementing volume was applied to further maximize the advantageous effect of FF.

Compared with CON, FF improved the milk yield, fat and protein contents and reduced the milk urea N and SCC of lactating sows. In contrast with the CON, PRO greatly increased the milk protein and decreased the milk urea N and SCC. However, milk lactose has no difference between the 3 treatments. Song *et al*. (2017) also revealed notably improved milk production, milk fat and protein when sow’s diets were supplemented with a fermented sorghum dried distiller’s grains with solubles. Additionally, fermented rapeseed positively influenced sows’ nutrient digestibility during lactation (Grela *et al*., 2019). According to Alexopoulos *et al*. (2004), *Bacillus* spp. significantly improved sow’s ADFI and milk quality. Ayala *et al*. (2015) found that *B. subtilis* is capable of promoting milk protein content of lactating sows. Sow’s milk yield and quality were assumed to be critical to piglets’ growth performance and affected by sow’s nutritional status (Kim *et al*., 2000). Consequently, the FF‐induced increase in greater ADFI and digestibility increase milk yield and quality, thereby improving the performance of the piglets. This improvement may be partially attributed to the probiotics of FF. Additionally, serum prolactin was improved in both FF and PRO groups. Serum prolactin can promote mammary gland development and improve lactation performance (Kleinberg and Ruan, 2008). Thus, the serum hormone level may contribute to milk yield and quality of sows. Urea N indicated the protein utilization of sows. The level of milk SCC is a predictive indicator of intramammary infected cows (Jashari *et al*., 2016). Hence, lower urea N and SCC in FF and PRO groups demonstrated better protein anabolic capacity and lower mastitis compared with the CON.

The serum immune parameters and SCFAs reflect the physiological and immune status of sows during the sensitive period of lactation. Serum immunoglobulin content is an important part of the sow immune system. Fermented feed increased serum IgM, IgG and reduced serum IgA content. Probiotic treatment showed a tendency to improve serum IgG content. Low serum IgA suggests a low risk of allergic reaction during lactation (Hansen *et al*., 2017). IgM is a vital anti‐inflammatory component and elevated IgM content indicated better immunity (Vaschetto *et al*., 2017). The increased concentration of IgG also reflected in a better immunological response and health for the lactating sows (Ayala *et al*., 2015). These results reveal a better immunoglobulin content of sows fed FF compared with that of CON. The lower TNF‐α concentrations and higher level of IL‐10 were observed for sows supplemented with FF in the present study, compared with CON. Serum TNF‐α and IFN‐γ are indicators of pro‐inflammatory reactions, while IL‐10 shows an anti‐inflammatory effect (Zhuo *et al*., 2020). Thus, the evidences of serum cytokines suggest FF prevents sows from potential inflammatory. Acetate and butyrate contents were greatly promoted in the FF group. Interestingly, the concentrations of SCFAs in PRO showed no obvious difference compared with other groups. Short‐chain fatty acids could enhance gut barrier functions, and butyrate can modulate immune response by inhibiting the pro‐inflammatory cytokine (D'souza *et al*., 2017). Collectively, FF balance sows’ immune status by regulating serum immunoglobulin and cytokines. Fermented feed showed greater immune modulating capacities than PRO in the present study. One possible explanation is an improvement of microbial metabolites, like SCFAs in the FF group, particularly acetate and butyrate.

The diversity of gut microbiota (Chao1 index) revealed a significant increase in the PRO group (*P* = 0.0014, Fig. 2A) and tented to increase in FF group. Increased microbial diversity contributes to a better gut condition and physiological preparation for the next production (Ji *et al*., 2019). Therefore, sows in FF and PRO group may had a better status for next production in contrast with CON. *Enterobacteriaceae* significantly decreased in both the FF‐ and P‐treated groups (*P* < 0.05, Fig. 2D). Other pathogens like *Klebsiella* also downregulated in FF‐treated groups but it did not significantly decrease in PRO group (*P* < 0.05, Fig. 2E). These results are consistent with previous studies (Urlings *et al*., 1993; Van Winsen *et al*., 2002; Price *et al*., 2010). The major pathogen in pig farms is *Enterobacteriaceae*, which can colonize the gut mucosa, resulting in damaged barrier functions and malnutrition (Mollenkopf *et al*., 2018). Low pH and probiotics from FF can prevent the infections of pathogens and enhance gut health effects (Missotten *et al*., 2015). Thus, organic acids and probiotics in FF in the present study positively modulated the sow gut bacteria and benefit to gut health. Probiotics like *Lactobacillus* and *Succiniclasticum* were dramatically promoted in the FF groups (*P* < 0.05, Fig. 2F). *Lactobacillus* is well studied for enhancing gut barrier functions, balancing gut microbiota, modulating innate immune systems and preventing of pathogen colonization, thereby benefiting to host health (Valeriano *et al*., 2017). *Succiniclasticum* spp. can convert succinate to propionate and beneficial to animal health (van Gylswyk, 1995). These results demonstrated that FF not only inhibits pathogens, but also plays important roles on gut probiotic modulating. Interestingly, supplementing probiotics alone only enriched the general level of *Synergistes* and *Mitsuokella*. The different effects of FF and PRO on sow gut microbiota, might due to the abundant organic acids and prebiotics provided from the FF. The network demonstrated that correlations of gut microbiota of sows were modulated by FF indicated by fewer cross‐linking and shorter interactions, which suggests a better relationships of gut microbiota (Zhou *et al*., 2019). In general, FF modulated sow’s gut microbiota by decreasing enrichment of pathogens and improving the abundance of *Lactobacillus* and *Succiniclasticum*.

The metabolic functional changes in the gut microbiota of lactating sows fed with FF or inoculated PRO are reported for the first time. The results showed that amino acid metabolism increased in the CON, which may be attributed to a high level of pathogens like *Enterobacteriaceae* and *Klebsiella* (Zhou *et al*., 2018). Lower amino acid metabolism in the FF group also indicates that protein profiles in FF‐included diet were more easily utilized by sows’ gut bacteria compared with that in CON. DNA repair and recombination proteins, glycolysis and gluconeogenesis, mismatch repair and d‐alanine metabolism were significantly upregulated in the FF group. FF may promote the change of gut microbial communities to greater replication and repair. As expected, PRO had different microbial functions such as histidine metabolism and transcription machinery compared with the FF group. Probiotic metabolites in FF may contribute to the different microbial functions between FF and PRO groups. The mechanism of these changes of metabolic function might be attributed to the shift of the gut microbiota.

The correlation analysis showed relationships between sows’ gut microbiota, feed characteristics and sows’ performance, demonstrating an underlying effect of gut microbiota affected by dietary FF and PRO on sow performance and immune status. Notably, the results revealed that *Lactobacillus* and *Succiniclasticum* were positively related with FF‐associated pH and small peptides and modulated the performance of sows and piglets, serum indexes and SCFAs. Feeding fermented rapeseed meal positively affected sow gut microbiota and improved the sow and offspring performance (Grela *et al*., 2019). Those results demonstrate that FF can benefit to sows’ gut microbiota, thereby improving their performance. Dietary probiotics including *Bacillus licheniformis* and *B. subtilis* spores (Alexopoulos *et al*., 2004), *E. faeciu*m NCIMB 10415 (Bohmer *et al*., 2006) were found to promote the physiological status and performance of lactating sows. These evidences reveal PRO partially contributes to the effects of FF. The FF‐enriched *Lactobacillus* and *Succiniclasticum* provide insights into strategies for improving the health and performance of lactating sows.

In summary, this study demonstrates the beneficial effects of *B. subtilis* and *E. faecium* co‐fermented feed on the performance, immune status and gut microbiota of lactating sows and firstly clarifies probiotics are not the only one effective factors in FF, which expanding the knowledge of underlying mechanism of FF’s beneficial effects. Later studies should further determine the most effective factors in the complex compounds of FF and its mechanism on modulating swine performance and health.

## Experimental procedures

### Fermented feed production

*Bacillus subtilis* CW4 (NCBI Accession No. MH885533) was discovered from pickled vegetables. *Enterococcus faecium* CWEF (NCBI Accession No. MN038173) was obtained from the intestinal of a healthy Jinhua pig. 40% of corn, 40% of soybean meal and 20% of yellow wine lees compose a basal substrate which was supplemented sterile water for the ultimate 40% moisture content. *B. subtilis* (1 × 10^8^ CFU g^−1^) and *E. faecium* (2 × 10^7^ CFU g^−1^) were added in the mixed substrate and fermented for 96 h. The nutrition determination of the UF and FF is shown in Table 1.

### Experimental design

All the procedures were approved by the Institutional Animal Care and Use Committee at Zhejiang University.

452 sows (Yorkshire × Landrace) were stochastically subjected to the following 3 treatments: (1) a control diet from a week before parturition to 21‐day weaning (*n* = 151), (2) a basal diet added with 10% of FF (*n* = 153) and (3) a basal diet added with the same amount of *B. subtilis* and *E. faecium* (*n* = 148). In the FF group, 10% of FF and 3% of soy oil took the place of 10% of corn and 3% of extruded SBM. For PRO, 3.5 g kg^−1^ of *Bacillus subtilis* powder and 1.2 g kg^−1^ of *Enterococcus faecium* powder were added in water to achieve the same amount of probiotics as FF. The diets contained equal CP and digestive energy (DE) concentration and meet the NRC (2012) nutrient requirement. Pelleted feed was used in all diets. The diet ingredients and nutrients are presented in Table 3.

**Table 3:** Ingredient composition and nutrient concentration in the experimental (as‐fed basis).^a^

Item	Diet		
	Control	10% FF	Probiotics
Ingredients, %			
Corn	60	50	60
Soybean meal, dehulled	10.0	7.0	10.0
Extruded soybean	14.0	14.0	14.0
Alfalfa meal	3.0	3.0	3.0
Fish meal	3.0	3.0	3.0
Soy oil	–	3	–
FF	–	10	–
Yeast hydrolysate	3.8	3.8	3.8
Citric acid	–	–	–
Baking soda	0.2	0.2	0.2
Salt	0.40	0.40	0.4
Limestone	0.6	0.6	0.6
Premix^b^	5.0	5.0	5.0
Total	100.00	100.00	100.00
Nutrition composition			
GE, MJ kg^−1^	15.89	15.97	15.83
DM, %	88.05	87.32	87.45
CP, %	17.36	17.45	17.72
EE, %	4.90	5.11	5.05
Ash, %	6.55	6.57	6.63
Ca, %	0.92	1.03	0.97
Total P, %	0.50	0.49	0.49
pH	6.04	5.58	5.96
Small peptides	3.7	7.09	3.88
Live BS cells, CFU g^−1^	–	3.2 × 10^4^	3.5 × 10^4^
Live EF cells, CFU g^−1^	–	1.4 × 10^4^	1.2 × 10^4^

BS = *Bacillus subtilis*; EE = ether extract; EF = *Enterococcus faecium*; FF = fermented feed.

^a^ Analysed values determined in duplicate.

^b^ Provided quantities of the following vitamins per kilogram of the complete diet: 10 000 IU vitamin A as vitamin A acetate, 1500 IU vitamin D_3_ as d‐activated animal sterol, 50 IU vitamin E as alpha tocopherol acetate, 4.4 mg vitamin K_3_ as menadione dimethylpyrimidinol bisulfite, 3.0 mg thiamin as thiamine mononitrate, 6.0 mg riboflavin, 3.0 mg pyridoxine as pyridoxine hydrochloride, 0.04 mg vitamin B_12_, 23 mg d‐pantothenic acid as calcium pantothenate, 36 mg niacin, 0.8 mg folic acid, 0.15 mg biotin and 186 mg choline as choline chloride. Also provided the following quantities of minerals per kilogram of the complete diet: 50 mg Cu as copper sulfate, 80 mg Fe as ferrous sulfate, 0.30 mg I as potassium iodate, 20 mg Mn as manganese sulfate, 0.2 mg Se as sodium selenite and 95 mg Zn as zinc sulfate.

The sows were randomly housed in farrowing crates on day 107 of gestation and the feed suppled was gradually increased during the perinatal period until feeding ad libitum. After delivery, the total number of born, live born and the birthweight of piglets were recorded immediately. After 21‐day weaning, the number of survivals and the weaning weight of piglets were recorded immediately. The sows’ feed intake during the experiment was measured. Sows’ backfat was determined following our previous study (Wang *et al*., 2018a). The constipation rate of sows and diarrhoea incidence of piglets were measured from parturition to weaning.

### Nutritional analyses

Diet samples from UF and FF were ground finely and then subjected to nutritional analysis. All samples were determined for gross energy (GE), dry matter (DM), CP, EE, ash, NDF, ADF, amylose, calcium (Ca) and total P contents (AOAC, 2005). Trichloroacetic acid‐soluble protein was determined as described by Ovissipour *et al*. (2009). The contents of antigenic protein were measured using an ELISA kit (Longke Ark Biological Engineering Technology, Beijing, China).

Six sows per group were randomly selected for determining milk yield and composition, serum indexes, faecal SCFAs and gut microbiota. 10 ml of blood from ear vein collected on day 21 of lactation was sampled to analyse hormone, immunoglobulins and cytokine concentrations. Blood samples were centrifuged and then stored at −20°C before determination. Fresh internal faecal samples of sows were collected to avoid contamination and obtain the most typical samples, then quickly transferred to 2.0 ml cryogenic vials (Sigma‐Aldrich^®^, Los Angeles, CA, USA), quick‐frozen in liquid nitrogen and placed at −80°C.

Milk yield was measured based on the method described by Kiers *et al*. (2003) and slightly modified. Briefly, the litter weights were measured for 6 continuous hours before and after suckling on day 21 after parturition. Milk yield per day = 24 × ∑(litter weight after suckling − litter weight before suckling)/6.

On day 21, approximate 40 ml of milk of six sows per group was randomly collected and placed at −20°C before analysis. The protein, fat, sugar, solids, urea N and SCC were used as a nutritional marker to assess the quality of milk by applying an automatic Milk Composition Analyzer (Shanghai, China).

The serum indexes were determined using a porcine immunoglobulin and cytokines kit (Jiangsu Meibiao Biological Technology, Jiangsu, China).

The SCFA contents were determined as follows: Briefly, 0.5 g of stool sample blended in 1.5 ml of distilled water. Afterwards, the sample was centrifuged (15 000 × *g*, 4°C, 20 min) and removed the 0.9 ml supernatant that mixed with a 0.1‐ml 25% (w/v) phosphoric acid, and stored at 4°C for 3 h. The sample then was centrifuged (10 000 × *g*) at 4°C for 10 min, filtered and submitted for gas chromatography (Varian CP‐3800 GC, USA).

### 16S sequencing and bioinformatics analyses

Briefly, the sample and sterile zirconium beads (0.3 g) were transferred to a 5 ml tube after thawing. The mixture was vigorously vortexed for 30 s after adding the 4.5 ml of TN150 buffer and then centrifuged at 200 × *g* for 5 min at 4°C. Then, the upper phase of the sample mixture (1 ml) was discharged. The pellet was retained and resuspended in 1 ml TN150 buffer, followed by centrifugation in an Eppendorf 5430 R microcentrifuge (Eppendorf, Berlin, Germany) at 100 × *g* for 5 min. After purification via phenol–chloroform (25 : 1) extraction, DNA was precipitated at −20°C for 4 h and dissolved in 60 µl nuclease‐free TE buffer. The concentration was detected in a NanoDrop One/Oneᶜ system (Thermo Fisher Scientific, Wilmington, NC, USA), and 2% of agarose gel electrophoresis (Biowest, Madrid, Spain) was performed to assess DNA purity. The DNA sample was then diluted to the proper concentration.

The composition of the bacterial community in faecal samples was characterized using the V4 region of 16S rRNA gene amplicon sequencing with primers (F: 5ʹ‐GTGCCAGCMGCCGCGG‐3ʹ and R: 5ʹ‐GGACTACHVGGGTWTCTAAT‐3ʹ). PCR amplifications were conducted according to the following parameters: 3 min of 95°C denaturation, followed by 27 cycles of 30 s at 95°C, annealing at 55°C for 30 s, and elongation at 72°C for 45 s, with a final extension at 72°C for 10 min. The amplification reactions were conducted three times in a 20 μl reaction system. The final products were extracted from 2% agarose gels and then purified with the MinElute Gel Extraction Kit (Qiagen, Dusseldorf, Germany) and assessed on an Invitrogen Qubit 4 system (Thermo Fisher, USA).

Before the preparation of the library, the PCR products were mixed and purified by using the GenElute™ PCR Clean‐Up Kit (Sigma, Los Angeles, CA, USA). The construction of sequencing libraries was conducted by using a DNA Seq Library Preparation Kit (Illumina, Madison, USA). Digital PCR Library Quantification Kit (Bio‐Rad, Irvine, CA, USA) and Qsep series Bio‐Fragment Analyzers (BiOptic, Taiwan, China) were used to assess the quality of the library. The Illumina MiSeq PE 300 platform was applied for sequencing to generate paired‐end reads (2 × 300 bp) from the sequence library based on a standard protocol.

### Bioinformatics analyses

The sequencing data analysis was performed based on the Quantitative Insight Into Microbial Ecology 1 (QIIME1, version 2.0, http://qiime.org/) analysis (Caporaso *et al*., 2010). An OTU table was generated from the effective tags that did not contain non‐biological nucleotides. The most abundant sequence in each OTU was regarded as the most representative sequence and used it for taxonomic annotation at the phylum to species levels against the greengene 13.5 database with 97% sequence identification based on the Ribosomal Database Project classifier (http://sourceforge.net/projects/rdp‐classifier/) (Wang *et al*., 2007). α and β diversities of the bacterial community were calculated by an online microbiome data analysis platform in the MicrobiomeAnalyst (http://www.microbiomeanalyst.ca/) based on Chao1 and unweighted UniFrac distance (Chong *et al*., 2020). The phylogenetic relationships among distinct OTUs and correlation plots were generated by R studio corrplot package. Furthermore, the predicted microbial metabolic functions were conducted by PICRUSt (https://huttenhower.sph.harvard.edu/galaxy/).

### Calculation and statistical analysis

The statistical analyses were performed by SPSS software (SAS, Chicago, IL, USA) using one‐way ANOVA analysis and Duncan’s multiple tests to compare the statistical differences. The differences between the three groups were defined as statistical significance at *P* < 0.05 and defined as trends at *P* < 0.10.

## Conflict of interest

The authors declare that there is no conflict of interest.
